# Host-adapted metabolism and its regulation in bacterial pathogens

**DOI:** 10.3389/fcimb.2015.00028

**Published:** 2015-03-27

**Authors:** Thomas Dandekar, Wolfgang Eisenreich

**Affiliations:** ^1^Bioinformatics, University of WürzburgWürzburg, Germany; ^2^Biochemistry, Technische Universität MünchenGarching, Germany

**Keywords:** bacterial pathogens, enteric pathogens, metabolism, host-pathogen adaption, isotopolog profiling

The mutual interaction between bacterial pathogens and their host organisms is a key feature of virulence. Since bacterial pathogens are highly specific in their infection behavior against different host organisms, cell types and cell compartments, the host-pathogen interplay is crucial in bacterial virulence. There is increasing evidence that basic metabolic pathways and fluxes in pathogens are modulated during the infection process as a response to adapt to the specific and dynamic conditions of the host environments, such as various cell types and compartments. Although a manipulation of these adaptation processes could be a key in the future treatment of bacterial infections, our knowledge about these metabolic features and their controlling factors is still limited.

Hence, the German research council (DFG) funded priority programme SPP1316 (2008–2014) for the investigation of host-adapted metabolism of bacterial pathogens. Within the SPP1316, a number of groups have investigated in considerable detail how bacterial pathogens adapt their metabolism during colonization of their host organisms, how the metabolism of pathogenic bacteria and host organism is interconnected and which mechanisms of control are active.

As a result for these investigations and further studies, various chapters of this “Topic hosted by Frontiers” report on the identification of metabolic pathways that are important for the bacterial pathogen during infection, and on determination of metabolic fluxes. Questions that are addressed by the Topic include: Why are bacteria able to multiply in the body of the host and can cause disease? Which specific toxins and/or invasins are produced? In general, which metabolic adaptations allow bacterial pathogens to colonize their host? Further points that are considered concern the interconnected metabolism of host and pathogen and the regulatory mechanisms that are activated in both pathogen and host. Furthermore, we cover several highly sensitive methods of bioanalytics and high throughput screening approaches developed in recent years. A systematic synopsis of metabolism of bacterial pathogens adapted to host conditions is given here.

Further chapters consider main metabolic pathways and fluxes, metabolic reactions of specific pathogens in their respective host niches as well as genetic and regulatory mechanisms of their metabolic adaptation. As a result, an integrated view on the nature and mechanisms in host-adapted bacterial metabolism is represented by the various articles of this Topic. The data show that host-pathogen metabolic adaptation is highly specific at least for most of the pathogens described in the Topic. Nevertheless, the data also demonstrate that the metabolic networks encountered in host-pathogen systems include essential reactions that could serve as future targets in drug therapy. It can be envisaged that further studies will foster our knowledge about metabolic features in host-adaptation of bacterial pathogens but also vice versa in the metabolic response of host organisms upon bacterial infections.

In summary, the Topic presents an integrated picture of the host- and pathogen adaptation of bacterial metabolism during infection, a bioanalytical characterization of the relevant metabolic pathways as well as insights by functional and analytical investigations, covering both extracellular and intracellular pathogens. We therefore believe that this research Topic will contribute in stimulating further studies in the fascinating field of host-pathogen interactions.

We present the following articles (blue font indicates articles of SPP1316 members)

## General aspects of host-adapted metabolism of bacterial pathogens

1. Metabolic aspects of bacterial persisters
  Marcel Prax and Ralph Bertram^*^
2. Interrelationship between type three secretion system and metabolism in pathogenic bacteria
  Gottfried Wilharm^*^ and Christine Heider
3. Small RNA functions in carbon metabolism and virulence of enteric pathogens
  Kai Papenfort^*^ and Jörg Vogel
4. From screen to target: insights and approaches into the development of anti-virulence compounds
  Katherine SH Beckham and Andrew J Roe

## Metabolism of intracellular bacterial pathogens

5. Analysis of carbon substrates used by *Listeria monocytogenes* during growth in J774A.1 macrophages suggests a bipartite intracellular metabolism
  Stephanie Grubmüller, Kristina Schauer, Werner Göbel, Thilo M Fuchs and Wolfgang Eisenreich
6. Nutrient generation and retrieval from the host cell cytosol by intra-vacuolar *Legionella pneumophila*
Christopher T. D. Price, Ashley Marie Richards and Yousef Abu Kwaik^*^
7. *Salmonella*—how a metabolic generalist adopts an intracellular life style during infection
  Thomas Dandekar^*^, Astrid Fieselmann, Eva Fischer, Jasmin Popp, Michael Hensel and Janina Noster
8. Metabolism of the vacuolar pathogen *Legionella* and implications for virulence
  Christian Manske and Hubert Hilbi
9. The *Mycobacterium avium* ssp. *paratuberculosis* specific *mptD* gene is required for maintaining of the metabolic homeostasis necessary for full virulence in mouse infections Thorsten
  Meißner, Elke Eckelt, Tina Basler, Jochen Meens, Julia Heinzmann, Aabdulhadi Suwandi, Walter Martin Roland Oelemann, Sandra Trenkamp, Otto Holst, Siegfried Weiss, Boyke Bunk, Cathrin Spöer, Gerald-F. Gerlach and Ralph Goethe

## Metabolism of extracellular bacterial pathogens

10. Coregulation of host-adapted metabolism and virulence by pathogenic yersiniae
  Ann Kathrin Heroven and Petra Dersch^*^
11. Defining the metabolic requirements for the growth and colonization capacity of *Campylobacter jejuni*
  Dirk Hofreuter^*^
12. *Staphylococcus aureus* small colony variants show common metabolic features in central metabolism irrespective of the underlying auxotrophism
  André Kriegeskorte^*^, Stephanie Grubmüller, Claudia Huber, Barbara C. Kahl, Christof von Eiff, Richard Allan Proctor, Georg Peters, Wolfgang Eisenreich and Karsten Becker^*^
13. Metabolism and virulence in *Neisseria meningitides*
  Christoph Schoen^*^, Laura Kischkies, Johannes Elias and Biju Joseph Ampattuu
14. The arginine-ornithine antiporter ArcD contributes to biological fitness of *Streptococcus suis*
  Marcus Fulde, Joerg Willenborg, Claudia Huber, Angela Hitzmann, Daniela Willms, Maren Seitz, Wolfgang Eisenreich, Peter Valentin-Weigand^*^ and Ralph Goethe
15. *Staphylococcus aureus* Small Colony Variants (SCVs): A road map for the metabolic pathways involved in persistent infections
  Richard Allan Proctor^*^, Andre Kriegeskorte, Barbara Kahl, Karsten Becker, Bettina Löffler and Georg Peters
16. *Yersinia pestis*: mechanisms of entry into and resistance to the host cell
  Yuehua Ke^*^, Zeliang Chen and Ruifu Yang

### Conflict of interest statement

The authors declare that the research was conducted in the absence of any commercial or financial relationships that could be construed as a potential conflict of interest.

